# Removal of the Uterus for Cancer

**Published:** 1881-12

**Authors:** 


### REMOVAL OF THE UTERUS FOR CANCER.

The November number of the "New York *Medical Journal and Obstetrical Review*" contains a "special article" by Dr. Andrew F.

Currier, of New York, in which the various methods of removing the entire uterus for cancer, as practiced by Freund, Schrœder, Czerny, and others, are reviewed, as well as the general question of the advisability of removing the organ. He thinks the advantages of the vaginal method over that of Freund (by laparotomy) are enormous—there is but one section of the peritonæum, the intestines are unharmed, there is a better opportunity to discover diseased tissue, which is most likely to be situated in the vicinity of the cervix, and, most important of all, the patients often survive, which is rare by Freund's method. But most patients are not likely to be benefited by either of these serious operations; the most hopeful cases will be those in which the patients are warned of their danger in the early stages of the disease, and in such cases Schrœder's supra-vaginal excision of the entire cervix is most likely to prove of service. This operation, while not so radical as removal of the entire organ, and hence not so efficient in cases involving the tissues above the internal os, is far less grave, and is, besides, more thorough than amputation of the cervix as it has ordinarily been done in the past. In those rare cases, however, in which the body of the uterus alone is involved, there is no alternative to laparotomy, either by Freund's operation or by some modification of it. As to drainage—a most important item in such cases—a perfect system seems impossible, but Bardenheuer's, although in the hands of others it has not fulfiled its author's expectation, affords as good results as any yet devised. As to the broad question of whether cancer of the uterus, and so cancer in general, can be radically cured, the author thinks the logic of events points to its approaching solution.

